# Reduced and Normalized Carbohydrate Antigen 19-9 Concentrations after Neoadjuvant Chemotherapy Have Comparable Prognostic Performance in Patients with Borderline Resectable and Locally Advanced Pancreatic Cancer

**DOI:** 10.3390/jcm9051477

**Published:** 2020-05-14

**Authors:** Woohyung Lee, Yejong Park, Jae Woo Kwon, Eunsung Jun, Ki Byung Song, Jae Hoon Lee, Dae Wook Hwang, Changhoon Yoo, Kyu-pyo Kim, Jae Ho Jeong, Heung-Moon Chang, Baek-Yeol Ryoo, Seo Young Park, Song Cheol Kim

**Affiliations:** 1 Division of Hepatobiliary and Pancreatic Surgery, Department of Surgery, University of Ulsan College of Medicine, Asan Medical Center, Seoul 05505, Korea; ywhnet@amc.seoul.kr (W.L.); blackpig856@gmail.com (Y.P.); skunlvup@naver.com (J.W.K.); jeongo1040@gmail.com (E.J.); mtsong21c@naver.com (K.B.S.); hbpsurgeon@gmail.com (J.H.L.); dwhwang@amc.seoul.kr (D.W.H.); 2 Department of Oncology, Asan Medical Center, University of Ulsan College of Medicine, Asan Medical Center, Seoul 05505, Korea; cyoo.amc@gmail.com (C.Y.); kkp1122@gmail.com (K.-p.K.); imdrho@gmail.com (J.H.J.); changhm@amc.seoul.kr (H.-M.C.); ryooby@amc.seoul.kr (B.-Y.R.); 3 Department of Clinical Epidemiology and Biostatistics, University of Ulsan College of Medicine, Asan Medical Center, Seoul 05505, Korea; biostat81@amc.seoul.kr

**Keywords:** pancreatic cancer, neoadjuvant chemotherapy, response, carbohydrate antigen 19-9

## Abstract

Background: The association between optimal carbohydrate antigen (CA) 19-9 concentration after neoadjuvant chemotherapy (NACT) and prognosis has not been confirmed in patients with borderline resectable (BRPC) and locally advanced pancreatic cancer (LAPC). Methods: This retrospective study included 122 patients with BRPC and 103 with LAPC who underwent surgery after NACT between 2012 and 2019 in a tertiary referral center. Prognostic models were established based on relative difference of the CA 19-9 (RDC), with their prognostic performance compared using C-index and Akaike information criterion (AIC). Results: CA 19-9 concentrations of 37–1000 U/mL before NACT showed prognostic significance in patients with BRPC and LAPC (hazard ratio [HR]: 0.262; 95% confidence interval [CI]: 0.092–0.748; *p* = 0.012). Prognostic models in this subgroup showed that RDC was independently prognostic of better overall survival (HR: 0.262; 95% CI: 0.093–0.739; *p* = 0.011) and recurrence free survival (HR: 0.299; 95% CI: 0.140–0.642; *p* = 0.002). The prognostic performances of RDC (C-index: 0.653; AIC: 227.243), normalization of CA 19-9 after NACT (C-index: 0.625; AIC: 230.897) and surgery (C-index: 0.613; AIC: 233.114) showed no significant differences. Conclusion: RDC was independently associated with better prognosis after NACT in patients with BRPC or LAPC. Decreased CA19-9 after NACT was a prognostic indicator of better survival and recurrence, as was normalization of CA 19-9 after both NACT and surgery.

## 1. Introduction

Pancreatic ductal adenocarcinoma (PDAC) is a rare gastrointestinal cancer, with patients having a dismal prognosis. Surgical treatment is the mainstay for curative treatment. However, only 15–20% of diagnosed patients have resectable disease, and only 30% have borderline resectable disease [1]. Radical surgery, including vascular reconstruction, has been reported as technically feasible, expanding surgical indications for PDAC [2]. Moreover, recent studies showed that patients with borderline resectable (BRPC) or locally advanced pancreatic cancer (LAPC) who underwent surgery after neoadjuvant chemotherapy (NACT) had better survival outcomes than those who underwent upfront surgery [3,4,5,6].

Although the optimal NACT regimen has not yet been determined, a recent meta-analysis found that FOLFIRINOX-based NACT yielded better oncologic outcomes than gemcitabine-based NACT, despite the former having greater toxicity [1,7]. The resection rate after NACT was 65.3%, with 57.4% of the patients who underwent surgery achieving R0 resection [7]. However, prognostic markers for responders to NACT have not yet been identified except circulating tumor cell or DNA [8]. Although several studies found that normalization of carbohydrate antigen (CA) 19-9 concentration is associated with better patient prognosis [9], 5–10% of patients with PDAC have normal CA 19-9 at diagnosis because of a Lewis-negative phenotype, and waiting until normalization of CA 19-9 is difficult in real-world practice [10,11]. This study investigated the ability of reduced CA 19-9 rather than normalized CA 19-9 after NACT to predict oncologic outcomes in patients with BRPC or LAPC. The present study also compared the prognostic ability of reduced and normalized CA 19-9 to evaluate response after NACT in patients with BRPC and LAPC.

## 2. Methods

### 2.1. Patients and Study Design

The present study included patients with BRPC and LAPC who underwent surgery following NACT at a tertiary referral center between July 2012 and August 2019. BRPC was defined as a tumor in contact with the common hepatic artery without extension to the celiac axis or hepatic artery bifurcation; a tumor in contact with ≤180° of the circumference of the superior mesenteric artery; a tumor in contact with >180° of the circumference of the superior mesenteric vein or portal vein; and a tumor in contact with ≤180° of the circumference of either vein and with a contour irregularity or thrombosis of the vein but with possible reconstruction [12]. Patients who underwent upfront surgery were excluded.

NACT was administered based on each patient‘s general condition, and concurrent radiotherapy was not used routinely. The patients were evaluated by serial abdominal computed tomography (CT) and positron emission tomography (PET), and by measuring CA 19-9 concentrations during NACT. CT was evaluated using modified Response Evaluation Criteria in Solid Tumors [13]. Surgery after NACT was evaluated by a multidisciplinary team based on regressive or stable tumor with possibility of resectability of involved vessels. Pathologic response after surgery was reported using the College of American pathologist regression grading system [14]. After operation, the patients were administered chemotherapy except those with complete resolution of PDAC. Radiotherapy was used in the patients with R1 resection. The response to chemotherapy after surgery was evaluated every 3 months for 2 years by means of abdominal CT and tumor markers. Recurrence was diagnosed based on serial imaging studies with changing tumor markers and biopsy if possible.

CA 19-9 concentrations were measured before and after NACT, and after surgery, with relative difference of the CA 19-9 (RDC) calculated as follows: [(CA19-9 after NACT) − (CA 19-9 before NACT)]/(CA 19-9 before NACT). The association between RDC and prognosis was investigated, and prognostic models were constructed for predicting overall survival (OS) and recurrence free survival (RFS). The abilities of normalized and reduced CA 19-9 concentrations to predict outcomes were compared.

Clinical data were obtained from patients’ medical records. Recorded preoperative factors included age, sex, body mass index (BMI), American Society of Anesthesiologists (ASA) score, and imaging results before and after NACT, with tumor markers. Intraoperative factors included extent of resection, operation time, intraoperative transfusion, and estimated blood loss. Pathologic factors included tumor regression grade, node metastasis, the number of retrieved lymph nodes, and the presence of lympho-vascular or perineural invasion. Postoperative factors included length of hospital stay, postoperative complication based on Clavien-Dindo classification, 30-day mortality, recurrence, and survival. Informed consent was obtained from each patient before surgery. The study protocol was approved by the Institutional Review Board of Asan Medical Center (IRB No: 2018-1336).

### 2.2. Statistical Analyses

Continuous variables are reported as the mean (standard deviation) and are compared by Student’s *t*-tests, and categorical variables are reported as numbers and percentages and are compared by χ^2^ tests. Survival rates were estimated by the Kaplan–Meier method and compared by log-rank tests. A multivariable Cox proportional hazards model was used to identify factors prognostic of OS and RFS. These variables were selected based on their clinical significance and statistical significance in a univariate Cox model, with caution to avoid overfitting and to ensure generalizability. To compare three methods of parameterization of CA19-9 (RDC, normalization after NACT, and postoperative normalization of CA19-9), c-indices were calculated for the final Cox model: one with RDC, the same model with RDC replaced by post-NACT normalization of CA 19-9, and the same model with RDC replaced by postoperative normalization of CA19-9. To evaluate the statistical difference of these three c-indices and Akaike information criterion (AIC), their standard errors were determined using 500 bootstrap samples, and their p-values were calculated. All statistical analyses were performed using SPSS^®^ version 22.0 (SPSS Corp., Chicago, IL, USA) and R 3.5.1 (R Foundation for Statistical Computing, Vienna, Austria) software, with two-sided *p*-values <0.05 considered statistically significant.

## 3. Results

### 3.1. Patient Characteristics

Gemcitabine or FOLFIRINOX based NACT were administered to 816 patients. Of these, 225 (27.5%) patients underwent curative intent surgery after NACT. A review of the medical records at our institution identified 225 eligible patients of mean age 59.7 years, including 115 (51.1%) men and 110 (48.9%) women. Their mean CA 19-9 concentrations before and after NACT were 676.5 U/mL and 188.4 U/mL, respectively, and their median RDC was 0.62 (with interquartile range: 0.21–0.85). Of these patients, 122 (54.2%) had BRPC and 103 (45.8%) had LAPC, with 96 (42.7%), 27 (12%), and 95 (42.3%) found to have invasion of the adjacent vein, artery, and both, respectively. The NACT regimen consisted of FOLFIRINOX-based chemotherapy in 167 (74.2%) patients and gemcitabine-based chemotherapy in 58 (25.8%), with 7 (3.1%) patients receiving concurrent radiotherapy. Surgery consisted of pancreaticoduodenectomy in 138 (61.3%) patients, distal pancreatectomy in 67 (29.8%), total pancreatectomy in 16 (7.1%), and palliative surgery in 4 (1.7%) depending on the tumor site, with 122 (54.2%) patients also undergoing adjacent vessel resection and 173 (76.9 %) undergoing R0 resection (Table 1).

### 3.2. Prognostic Implications of RDC Based on CA19-9 Concentration before NACT

**Figure 1:**
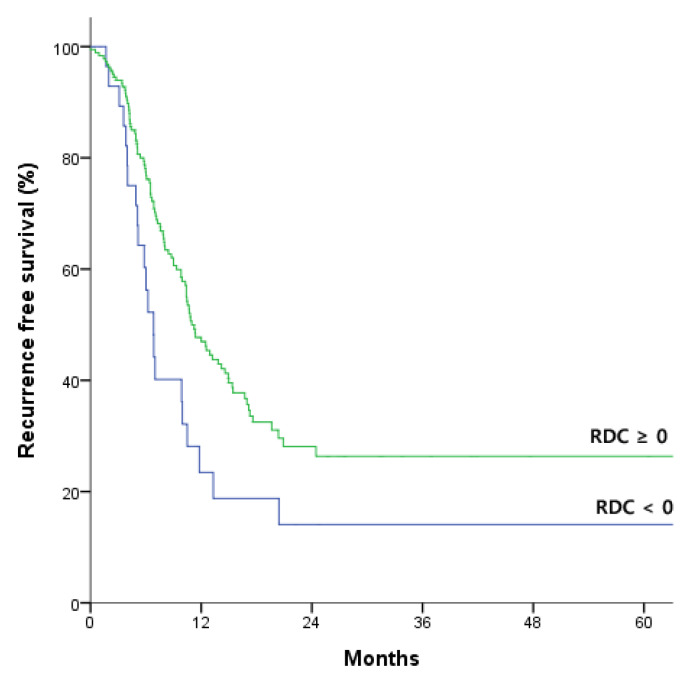
Kaplan-Meier analysis of recurrence free survival in patients with relative difference of carbohydrate antigen 19-9 (RDC) ≥ 0 and < 0. Median recurrence free survival was significantly longer in patients with RDC ≥ 0 than in those with RDC < 0 (10.9 vs. 6.8 months; *p* = 0.016 by log-rank test).

**Figure 2:**
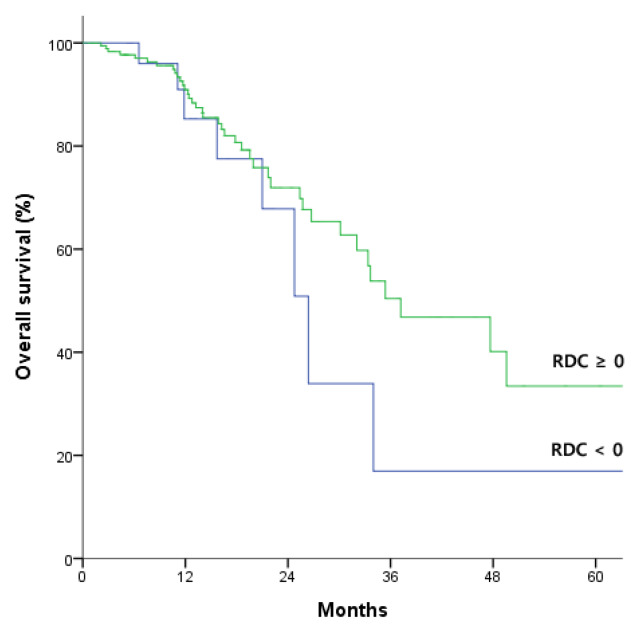
Kaplan-Meier analysis of overall survival in patients relative difference of carbohydrate antigen 19-9 (RDC) ≥ 0 and < 0. Median overall survival showed no significant difference between patients with RDC ≥ 0 and RDC < 0 (37.1 vs. 26.3 months; *p* = 0.293 by log-rank test).

Of 225 patients, 30 (13.3%) patients showed increased CA19-9 after NACT, which means RDC < 0, and 188 (83.6 %) patients showed decreased or similar CA19-9 after NACT, which means RDC ≥ 0. We compared oncologic outcomes between RDC ≥ 0 and RDC < 0 groups. The patients with RDC ≥ 0 showed better median recurrence free period compared with RDC < 0 significantly (10.9 vs. 6.8 months, *p* = 0.016; Figure 1). There was no significant difference in median survival period between RDC ≥ 0 and RDC < 0 groups (37.1 vs. 26.3 months, *p* = 0.293; Figure 2). Perioperative variables were compared between two groups. The patients with RDC < 0 showed higher nodal stage than RDC ≥ 0 group (*p* = 0.037). Otherwise, there were no significant differences between the two groups (Supplementary Table S1). We investigated the effect of prognosis based on CA 19-9 concentration before NACT and the degree of reduction during NACT. Normal and high CA 19-9 concentrations were defined as < 37 U/mL and >1000 U/mL, respectively, with concentrations of 37–1000 U/mL classified as intermediate [15]. Neither the 62 patients with CA19-9 < 37 U/mL nor the 26 patients with CA 19-9 >1000 U/mL before NACT showed significant improvements in OS and RFS during NACT. However, the 133 patients with CA 19-9 37–1000 U/mL before NACT showed better OS (hazard ratio [HR]: 0.262; 95% confidence interval [CI]: 0.092–0.748; *p* = 0.012) and RFS (HR: 0.290; 95% CI: 0.134–0.628; *p* = 0.002) after NACT. Because these CA 19-9 concentrations before NACT were prognostically significant of survival outcomes after NACT, we established prognostic models and compared their prognostic performance in this patient subgroup (Table 2).

### 3.3. Establishment of Prognostic Model for Survival and Recurrence in Patients with pre-NACT 37–1000 U/mL

The 3-year OS and RFS rates were 43.7% and 24.8%, respectively. Univariate analysis showed that adjacent vein resection (HR: 2.121; 95% CI: 1.028–4.377; *p* = 0.042), low RDC as a continuous variable (HR: 0.262; 95% CI: 0.092–0.748; *p* = 0.012), and intraoperative transfusion (HR: 2.172; 95% CI: 1.022–4.619; *p* = 0.044) were significantly associated with worse OS. Multivariate analysis showed that low RDC was the independent prognostic factor for worse OS (HR: 0.262; 95% CI: 0.093–0.739; *p* = 0.011; Table 3). Univariate analysis of factors prognostic of RFS found that adjacent vein resection (HR: 1.687; 95% CI: 1.075–2.649; *p* = 0.023), advanced T-stage (*p* = 0.013), and low RDC (HR: 0.290; 95% CI: 0.134–0.628; *p* = 0.002), were significantly prognostic of reduced RFS. Multivariate analysis showed that low RDC (HR: 0.299; 95% CI: 0.140–0.642; *p* = 0.002) and adjacent vein resection (HR: 1.612; 95% CI: 1.021–2.545; *p* = 0.040) were independently prognostic of early recurrence (Table 4).

### 3.4. Comparative Prognostic Performance of Reduced and Normalized CA 19-9 after NACT and after Surgery

We compared prognostic performance of model using factors related with prognosis in this study. Prognostic model for OS included adjacent vein resection, and intraoperative transfusion, Additionally, Model 1, 2, and 3 included RDC as a continuous variable, normalization of CA19-9 after NACT, and normalization of CA19-9 after surgery, respectively. Model 1, 2, and 3 had C-index values for OS of 0.653, 0.625, and 0.613, respectively. Although the C-index of model 1 was higher than those of models 2 and 3, the differences were not statistically significant (*p* = 0.904 and *p* = 0.680, respectively). The AIC values for OS of models 1, 2, and 3 were 227.243, 230.897, and 233.114, respectively, with no statistically significant differences between model 1 and models 2 (*p* = 0.896) and 3 (*p* = 0.912). Prognostic model for RFS included adjacent vein resection. Additionally, Model 1, 2, and 3 included RDC as a continuous variable, normalization of CA19-9 after NACT, and normalization of CA19-9 after surgery, respectively. The three models had C-index values for RFS of 0.604, 0.584, and 0.602, respectively, with no statistically significant differences between model 1 and models 2 (*p* = 0.812) and 3 (*p* = 0.592). The AIC values for RFS of models 1, 2, and 3 groups were 636.138, 640.246, and 638.247, respectively, with no statistically significant differences between model 1 and models 2 (*p* = 0.900) and 3 (*p* = 0.924). Thus, the prognostic performances of the three models for OS and RFS were similar (Table 5).

### 3.5. Prognostic Models in Patients with Borderline Resectable Pancreatic Cancer and Locally Advanced Pancreatic Cancer

We evaluated performance for the prognostic model in patients with CA19-9 concentration of 37–1000 U/mL before NACT for BRPC (*n* = 73) and LAPC (*n* = 60) subgroup. In BRPC subgroup, adjacent vein resection (HR: 2.806; 95% CI: 1.116–7.051; *p* = 0.028) and RDC (HR: 0.456; 95% CI: 0.211–0.986; *p* = 0.046) were independent prognostic factors for OS. RDC (HR: 0.279; 95% CI: 0.137–0.570; *p* < 0.001) was an independent prognostic factor for RFS. We evaluated prognostic performance among models including prognostic factors in BRPC subgroup. Model 1 (C-index; 0.680, AIC; 126.030), 2 (C-index; 0.693, AIC; 126.403), and 3 (C-index; 0.682, AIC; 128.347) for OS showed no statistical significance among groups. Prognostic model 1 (C-index; 0.624, AIC; 302.496), 2 (C-index; 0.647, AIC; 302.562), and 3 (C-index; 0.622, AIC; 314.809) for RFS showed similar results without statistical significance. In LAPC subgroup, Cell differentiation (HR: 86.399; 95% CI: 5.806–1285.760; *p* = 0.001) was an independent prognostic factor for OS. T stage (HR: 2.545; 95% CI: 1.060–6.110; *p* = 0.037), and RDC (HR: 0.634; 95% CI: 0.451–0.889; *p* = 0.008) were independent prognostic factors for RFS. Prognostic models for OS showed no statistical significance among model 1 (C-index; 0.919, AIC; 49.475), 2 (C-index; 0.907, AIC; 49.327), and 3 (C-index; 0.896, AIC; 49.476). Prognostic models for RFS showed no statistical significance among model 1 (C-index; 0.646, AIC; 193.838), 2 (C-index; 0.606, AIC; 199.314), and 3 (C-index; 0.661, AIC; 193.408) (Supplementary Table S2–S7).

## 4. Discussion

This study showed that a decrease in CA19-9 concentration after NACT was an indicator of better prognosis in patients with BRPC or LAPC. Furthermore, comparisons of three prognostic models of reduced and normalized CA 19-9 after NACT, and of normalized CA 19-9 after surgery, showed that the three models were similarly predictive of OS and RFS.

CA19-9 is a Lewis blood group oligosaccharide, also called sialyl Lewis A antigen, which is synthesized by exocrine epithelial cells. It has shown a 70–90% predictive value for diagnosing pancreatic cancer [16]. However, elevated CA 19-9 has also been associated with other gastrointestinal tumors, as well as with biliary tract inflammation. Moreover, 5–10% of patients with PDAC are Lewis antigen negative, with normal CA 19-9 concentrations [11]. CA 19-9 concentration after NACT may be a biologic marker in patients with BRPC and LAPC because normalized or reduced CA 19-9 concentration after NACT has been reported to be an important prognostic marker of better OS and RFS [17]. Compared with patients with RDC ≤ 0.5, those with RDC > 0.5 experienced better survival and higher resectability after NACT, suggesting that early surgery may benefit rapid responders [18]. However, 83% of responders had RDC > 0.5 after NACT; of these, 24% had resectable PDAC, suggesting they were biologically good responders. Although normalization of CA19-9 after NACT was found to be more prognostic of survival outcomes than reduced CA 19-9, that study included patients with CA 19-9 >1000 U/mL, with this subgroup showing higher CA19-9 and a lower normalization rate after NACT than patients with CA19-9 <1000 U/mL [9]. In addition, the evaluation of the relationship between RDC and OS in that study also included patients with high CA 19-9 concentrations. The present study found that RDC after NACT affected patient prognosis. High preoperative CA 19-9 was shown to be associated with early recurrence and lower resectability rates [16,19,20]. Patients with CA19-9 >1000 U/mL were classified as having BRPC, with NACT recommended even in patients with resectable tumors [15]. In the present study, only 14% of patients with high CA 19-9 before NACT achieved normalization after NACT, with survival outcomes being poorer than in patients with CA19-9 <1000 U/mL before NACT, although the differences were not statistically significant. Other markers are required to evaluate tumor response in this subgroup. By contrast, evaluation of response to NACT using CA19-9 is inadequate for patients with CA19-9 <37 U/mL. These patients may have a Lewis-negative phenotype, suggesting that other markers, such as CEA and CA125, are needed to check their biologic status [21]. However, CA 19-9 concentrations were found to be elevated in patients with pancreatic cancer, despite 27.4% of these patients being Lewis antigen negative, suggesting that CA19-9 may be helpful in diagnosing pancreatic cancer in Lewis-negative patients, except in those with extremely low CA19-9 ≤5 U/mL [10,21].

This study found that RDC was an independent prognostic factor and that survival outcomes were better in good responders. Similarly, a previous study showed that RDC > 0.5 was an independent predictor of OS and that RDC > 0.9 was associated with pathologic complete regression [18]. The present study also showed similar prognostic performances of reduced and normalized CA 19-9 after NACT. That is, prognosis was similar in patients with higher RDC after NACT and in patients with normalized CA 19-9 after NACT or surgery.

The present study also found that the change of CA 19-9 was unable to predict the need for resection of adjacent vessels, R0 resection, or tumor regression grade. RDC was not a biologic marker predictive of curative resection after NACT. Similarly, normalization of CA 19-9 was not associated with a histopathologic response, with a negative predictive value of 28% [22]. Furthermore, radiologic response was not related to histologic response [23,24,25]. Additional studies are needed to identify biomarkers of resectability after NACT [26,27].

This study had several limitations, including its retrospective design, which may have resulted in potential selection bias. Furthermore, the relatively small number of patients was another limitation. However, this disease entity is rare, indicating a need for multicenter studies to evaluate larger patient populations.

## 5. Conclusions

RDC was independently prognostic of better OS and RFS rates in patients with CA19-9 concentrations of 37–1000 U/mL prior to NACT. Although normalization of CA19-9 after NACT is an indicator of good patient prognosis, its prognostic performance was comparable to a decrease in CA 19-9 during NACT.

## Figures and Tables

**Table 1:** Patient characteristics (*n* = 225).

	*n* (%) or Mean ± SD
Age (years)	59.7 ± 8.6
Sex (M/F)	115 (51.1)/110 (48.9)
ASA score (I/II/III)	15 (6.7)/189 (84)/19 (8.4)
BRPC/LAPC	122 (54.2)/103 (45.8)
Invasion (SMV/SMA/Both)	96 (42.7)/27 (12)/95 (42.3)
NACT regimen	
Gemcitabine based	58 (25.8)
FOLFIRINOX based	167 (74.2)
NACT cycle	6.5 ± 3.3
Concurrent neoadjuvant radiotherapy	7 (3.1)
CA19-9 before NACT (U/mL)	676.5 ± 3142.3
CA19-9 after NACT (U/mL)	188.4 ± 522.1
Median relative change of CA19-9 during NACT	0.62 (interquartile range: 0.21–0.85)
CA19-9 7 days after surgery (U/mL)	166.0 ± 1500.4
Preoperative response on CT (PR/SD)	67 (29.8)/158 (70.2)
Operation time	315.2 ± 97.4
Operation (PD/DP/TP/Palliative surgery)	138 (61.3)/67 (29.8)/16 (7.1)/4 (1.7)
Intraoperative transfusion	37 (16.4)
Vessel resection (vein/artery)	95 (57.8)/41 (18.2)
Adjacent organ resection	19 (8.4)
Postoperative complication	44 (19.6)
Differentiation (CR/WD/MD/PD/UD)	5 (2.2)/26 (11.6)/172 (76.4)/16 (7.1)/2 (0.9)
T-stage (CR/1/2/3/4), AJCC 8th	5 (2.2)/63 (28.0)/124 (55.1)/29 (12.9)/4 (1.8)
N-stage (0/1/2), AJCC 8th	122 (54.2)/80 (35.6)/23 (10.2)
Resection margin (R0/R1)	173 (76.9)/48 (21.3)

SD, standard deviation; ASA, American Society of Anesthesiologists; BRPC, borderline resectable pancreatic cancer; LAPC, locally advanced pancreatic cancer; NACT, neoadjuvant chemotherapy; FOLFIRINOX, 5-fluorouracil, irinotecan, and oxaliplatin; CA 19-9, carbohydrate antigen 19-9; CT, computed tomography; PR, partial response; SD, stable disease; PD, pancreaticoduodenectomy; DP, distal pancreatectomy; TP, total pancreatectomy; CR, complete regression; WD, well differentiated; MD, moderately differentiated; PD, poorly differentiated; UD, undifferentiated; AJCC, American Joint Committee on Cancer.

**Table 2:** Prognostic effects of carbohydrate antigen 19-9 concentration before neoadjuvant chemotherapy on overall survival and recurrence free survival.

	CA19-9 before NACT	HR	95% CI	*p*-Value
Overall survival	<37 U/mL (*n* = 62)	0.851	0.100–7.241	0.882
	37–1000 U/m (*n* = 133)	0.262	0.092–0.748	0.012
	>1000 U/mL (*n* = 26)	8.075	0.163–399.699	0.294
Recurrence free survival	<37 U/mL (*n* = 62)	0.708	0.222–2.257	0.560
	37–1000 U/mL (*n* = 133)	0.290	0.134–0.628	0.002
	>1000 U/mL (*n* = 26)	1.016	0.211–4.888	0.985

HR, hazard ratio; CI, confidence interval; CA19-9, carbohydrate antigen 19-9; NACT, neoadjuvant chemotherapy.

**Table 3:** Univariate and multivariate analyses of factors associated with overall survival in patients with pancreatic cancer and carbohydrate antigen 19-9 concentrations of 37–1000 U/mL before neoadjuvant chemotherapy.

		Univariate Analysis			Multivariate Analysis		
Variables		HR	95% CI	*p*-Value	HR	95% CI	*p*-Value
Age		1.019	0.978–1.062	0.368			
Sex		0.884	0.434–1.801	0.734			
Partial response on preoperative CT		1.076	0.792–1.460	0.640			
Adjacent vein resection		2.121	1.028–4.377	0.042	1.923	0.897–4.122	0.093
Cell differentiation	WD,MD/PD,UD	3.202	1.243–8.245	0.016			
T-stage (AJCC 8th)	1,2/3,4	1.124	0.500–2.524	0.777			
N-stage (AJCC 8th)	N0 (ref)	1		0.560			
	N1	1.144	0.524–2.496	0.736			
	N2	1.806	0.649–5.021	0.257			
Tumor regression grade	0,1/2,3	1.139	0.403–3.219	0.806			
Lympho-vascular invasion		1.637	0.802–3.342	0.175			
Perineural invasion		1.306	0.531–3.209	0.561			
RDC		0.262	0.092–0.748	0.012	0.262	0.093–0.739	0.011
R1 resection		1.366	0.621–3.004	0.437			
Intraoperative transfusion		2.172	1.022–4.619	0.044	1.977	0.910–4.296	0.085

HR, hazard ratio; CI, confidence interval; CT, computed tomography; WD, well differentiated; MD, moderately differentiated; PD, poorly differentiated; UD, undifferentiated; AJCC, American Joint Committee on Cancer; RDC, relative difference of CA19-9.

**Table 4:** Univariate and multivariate analyses of factors associated with recurrence free survival in patients with pancreatic cancer and carbohydrate antigen 19-9 concentrations of 37–1000 U/mL before neoadjuvant chemotherapy.

		Univariate Analysis			MultivAriate Analysis		
Variables		HR	95% CI	*p*-Value	HR	95% CI	*p*-Value
Age		0.987	0.965–1.010	0.263			
Sex		1.161	0.745–1.808	0.509			
Partial response on preoperative CT		0.873	0.600–1.270	0.478			
Adjacent vein resection		1.687	1.075–2.649	0.023	1.612	1.021–2.545	0.040
Cell differentiation	WD,MD/PD,UD	1.184	0.577–2.429	0.198			
Tumor regression grade	0,1/2,3	1.531	0.800–2.930	0.198			
Lympho-vascular invasion		1.337	0.847–2.110	0.212			
Perineural invasion		1.261	0.751–2.118	0.381			
T-stage (AJCC 8th)	1,2/3,4	0.923	0.568–1.501	0.747			
N-stage (AJCC 8th)	N0 (ref)			0.159			
	N1	1.550	0.208–11.553	0.669			
	N2	1.883	0.984-3.607	0.056			
RDC		0.290	0.134–0.628	0.002	0.299	0.140–0.642	0.002
R1 resection		1.459	0.873–2.437	0.150			
Intraoperative transfusion		1.171	0.667–2.056	0.583			

HR, hazard ratio; CI, confidence interval; CT, computed tomography; WD, well differentiated; MD, moderately differentiated; PD, poorly differentiated; UD, undifferentiated; AJCC, American Joint Committee on Cancer; RDC, relative difference of carbohydrate antigen 19-9.

**Table 5:** Prognostic performance of models that included decreases and normalization of carbohydrate antigen 19-9 concentration after neoadjuvant chemotherapy and surgery on overall survival and recurrence free survival.

Outcome	Prognostic Model	C-Index	95% CI	*p*-Value (1 vs. 2)	*p*-Value (1 vs. 3)	AIC	95% CI	*p*-Value (1 vs. 2)	*p*-Value (1 vs. 3)
Overall survival	Model 1	0.653	0.530–0.784	0.904	0.680	227.243	142.091–297.964	0.896	0.912
	Model 2	0.625	0.501–0.767			230.897	145.695–303.928		
	Model 3	0.613	0.523–0.760			233.114	148.409–305.646		
Recurrence free survival	Model 1	0.604	0.534–0.676	0.812	0.592	636.138	546.638–726.578	0.900	0.924
	Model 2	0.584	0.532–0.654			640.246	548.808–727.833		
	Model 3	0.602	0.547–0.673			638.247	545.773–722.073		

CI, confidence interval; AIC, Akaike information criterion; Model 1, prognostic model including decreased carbohydrate antigen 19-9 concentration during neoadjuvant chemotherapy; Model 2, prognostic model including normalization of carbohydrate antigen 19-9 concentration after neoadjuvant chemotherapy; Model 3, prognostic model including normalization of carbohydrate antigen 19-9 concentration after surgery.

## Supplementary Materials

The following are available online at https://www.mdpi.com/2077-0383/9/5/1477/s1, Table S1: Characteristics of patients with relative difference of CA19-9 < 0 and ≥ 0, Table S2: Prognostic factors associated with overall survival in patients with borderline resectable pancreatic cancer and carbohydrate antigen 19-9 concentrations of 37–1000 U/ml before neoadjuvant chemotherapy (N = 73), Table S3: Prognostic factors associated with recurrence free survival in patients with borderline resectable pancreatic cancer and carbohydrate antigen 19-9 concentrations of 37–1000 U/ml before neoadjuvant chemotherapy (N = 73), Table S4: Prognostic performance of models that included decreases and normalization of carbohydrate antigen 19-9 concentration after neoadjuvant chemotherapy and surgery on overall survival and recurrence free survival for borderline resectable pancreatic cancer, Table S5: Prognostic factors associated with overall survival in patients with locally advanced pancreatic cancer and carbohydrate antigen 19-9 concentrations of 37–1000 U/ml before neoadjuvant chemotherapy (N = 60), Table S6: Prognostic factors associated with recurrence free survival in patients with locally advanced pancreatic cancer and carbohydrate antigen 19-9 concentrations of 37–1000 U/ml before neoadjuvant chemotherapy (N = 60), Table S7. Prognostic performance of models that included decreases and normalization of carbohydrate antigen 19-9 concentration after neoadjuvant chemotherapy and surgery on overall survival and recurrence free survival for locally advanced pancreatic cancer.
