# Supplementary material for: Reduced and Normalized Carbohydrate Antigen 19-9 Concentrations after Neoadjuvant Chemotherapy Have Comparable Prognostic Performance in Patients with Borderline Resectable and Locally Advanced Pancreatic Cancer
Source: J Clin Med. 2020 May 14;9(5):1477. doi: 10.3390/jcm9051477 (PMC7291310; doi:10.3390/jcm9051477)
Supplement: Supplementary file 1 [file jcm-09-01477-s001.pdf]

Supplementary

Table S1. Characteristics of patients with relative difference of CA19-9 < 0 and ≥ 0.

	**RDC < 0 (n = 30)**	**RDC ≥ 0 (n = 188)**	**p-value**
Age (years)	59.6 ± 9.9	59.6 ± 8.4	0.999
Female	13 (43.3)	96 (51.1)	0.432
ASA score (I / II / III)	0/26(86.7)/4(13.3)	14(7.5)/159(85.5)/13(7.0)	0.167
BRPC	15 (50)	101 (53.7)	0.781
Pre-NACT CA 19-9 (U/ml)	510.6 ± 889.2	710.5 ± 3388.8	0.749
Post-NACT CA 19-9 (U/ml)	750.7 ± 1141.7	101.3 ± 244.5	0.004
Post-OP CA 19-9 (U/ml)	346.9 ± 960.7	143.9 ± 1589.5	0.519
NACT regimen (FOLFIRINOX)	22 (73.3)	142 (75.5)	0.796
NACT with RTx	2 (6.7)	5 (2.7)	0.248
NACT cycle	7.0 ± 4.4	6.6 ± 3.1	0.508
Pre-OP CA 19-9 < 37 U/ml	6 (20)	114 (60.6)	<0.001
Pre-OP CT PR / SD	6 (20) / 24(80)	60 (31.9) / 128 (68.1)	0.187
Operation time (min)	334.3 ± 100.9	314.3 ± 95.2	0.385
Intraoperative transfusion	5 (16.7)	31 (16.5)	0.981
Vessel resection	16 (53.3)	102 (54.3)	0.925
Adjacent organ resection	1 (3.3)	17 (9.0)	0.291
Differentiation (WD, MD / PD, UD)	25 (89.3)/3(10.7)	160(93.6)/11(6.4)	0.412
Tumor size (cm)	2.7 ± 1.3	2.5 ± 1.4	0.596
N-stage (0 / 1 / 2), AJCC 8th	11 (36.7)/14(46.7)/5(16.7)	108(57.4)/62(33)/18(9.6)	0.037
Tumor regression grade (0, 1/2)	4 (14.8)/ 23 (85.2)	18 (10.0) /162(90.0)	0.499
Lymphovascular invasion	14 (50)	65 (35.1)	0.129
Perineural invasion	21 (75)	127 (68.6)	0.496
R1 resection	10 (34.5)	37 (19.9)	0.077
Postop complications	10 (33.3)	34 (18.1)	0.053
Hospital stay (days)	13.6 ± 10.7	13.2 ± 7.1	0.872
Adjuvant chemotherapy	23 (82.1)	150 (81.1)	0.893

RDC, relative difference of CA19-9.; ASA, American Association of Anesthesiologists; BRPC, borderline resectable pancreatic cancer; NACT, neoadjuvant chemotherapy; FOLFIRINOX, 5-fluorouracil, irinotecan, and oxaliplatin; CA 19-9, carbohydrate antigen 19-9; OP, operation; CT, computed tomography; PR, partial response; SD, stable disease; WD, well differentiated; MD, moderately differentiated; PD, poorly differentiated; UD, undifferentiated; AJCC, American Joint Committee on Cancer

Table S2. Prognostic factors associated with overall survival in patients with borderline resectable pancreatic cancer and carbohydrate antigen 19-9 concentrations of 37–1000 U/ml before neoadjuvant chemotherapy (N = 73).

		Univariate analysis			Multivariate analysis		
**Variables**		**HR**	**95% CI**	**p-value**	**HR**	**95% CI**	**p-value**
Age		1.017	0.963-1.074	0.536			
Sex		1.094	0.452-2.645	0.842			
Partial response on preoperative CT		0.671	0.277-1.621	0.375			
Adjacent vein resection		2.828	1.141-7.010	0.025	2.806	1.116-7.051	0.028
Cell differentiation	WD,MD/PD,UD	3.306	0.734-14.898	0.120			
T-stage (AJCC 8th)	1,2/3,4	2.163	0.713-6.558	0.173			
N-stage (AJCC 8th)	N0 (ref)	1		0.432			
	N1	0.937	0.353-2.482	0.895			
	N2	2.215	0.590-8.308	0.238			
Tumor regression grade	0,1/2,3	0.863	0.192-3.886	0.848			
Lymphovascular invasion		1.568	0.644-3.817	0.322			
Perineural invasion		1.358	0.490-3.762	0.556			
RDC		0.420	0.184-0.962	0.040	0.456	0.211-0.986	0.046
R1 resection		0.562	0.162-1.950	0.364			
Intraoperative transfusion		2.614	1.079-6.332	0.033	1.927	0.732-5.072	0.184

HR, hazard ratio; CI, confidence interval; CT, computed tomography; WD, well differentiated; MD, moderately differentiated; PD, poorly differentiated; UD, undifferentiated; AJCC, American Joint Committee on Cancer; RDC, relative difference of CA19-9.

Table S3. Prognostic factors associated with recurrence free survival in patients with borderline resectable pancreatic cancer and carbohydrate antigen 19-9 concentrations of 37–1000 U/ml before neoadjuvant chemotherapy (N = 73).

		Univariate analysis			Multivariate analysis		
**Variables**		**HR**	**95% CI**	**p-value**	**HR**	**95% CI**	**p-value**
Age		0.978	0.209-0.978	0.209			
Sex		0.854	0.471-1.548	0.602			
Partial response on preoperative CT		0.875	0.479-1.598	0.664			
Adjacent vein resection		1.804	0.991-3.285	0.054	1.786	0.965-3.307	0.065
Cell differentiation	WD,MD/PD,UD	1.783	0.538-5.906	0.344			
Tumor regression grade	0,1/2,3	2.043	0.491-8.504	0.326			
Lymphovascular invasion		1.078	0.582-1.995	0.812			
Perineural invasion		1.116	0.606-2.221	0.653			
T-stage (AJCC 8th)	1,2/3,4	3.366	1.482-7.645	0.004	1.629	0.556-4.770	0.374
N-stage (AJCC 8th)	N0 (ref)			0.203			
	N1	0.631	0.327-1.217	0.169			
	N2	1.475	0.559-3.896	0.432			
RDC		0.276	0.132-0.577	0.001	0.279	0.137-0.570	<0.001
R1 resection		1.301	0.654-2.587	0.453			
Intraoperative transfusion		1.269	0.663-2.431	0.472			

HR, hazard ratio; CI, confidence interval; CT, computed tomography; WD, well differentiated; MD, moderately differentiated; PD, poorly differentiated; UD, undifferentiated; AJCC, American Joint Committee on Cancer; RDC, relative difference of CA19-9.

Table S4. Prognostic performance of models that included decreases and normalization of carbohydrate antigen 19-9 concentration after neoadjuvant chemotherapy and surgery on overall survival and recurrence free survival for borderline resectable pancreatic cancer.

Outcome		C-index	95% CI	p-value (1 vs. 2)	p-value (1 vs. 3)	AIC	95% CI	p-value (1 vs. 2)	p-value (1 vs. 3)
Overall survival	Model 1	0.680	0.531-0.844	0.988	0.656	126.030	74.941-182.038	0.800	0.780
	Model 2	0.693	0.571-0.847			126.403	75.687-182.315		
	Model 3	0.682	0.552-0.832			128.347	75.899-181.795		
Recurrence free survival	Model 1	0.624	0.540-0.730	0.936	0.936	302.496	239.024-355.597	0.836	0.844
	Model 2	0.647	0.583-0.743			302.562	240.604-356.290		
	Model 3	0.622	0.549-0.709			314.809	256.086-366.281		

CI, confidence interval; AIC, Akaike information criterion; Model 1, prognostic model including decreased carbohydrate antigen 19-9 concentration during neoadjuvant chemotherapy; Model 2, prognostic model including normalization of carbohydrate antigen 19-9 concentration after neoadjuvant chemotherapy; Model 3, prognostic model including normalization of carbohydrate antigen 19-9 concentration after surgery.

Table S5. Prognostic factors associated with overall survival in patients with locally advanced pancreatic cancer and carbohydrate antigen 19-9 concentrations of 37–1000 U/ml before neoadjuvant chemotherapy (N = 60).

		Univariate analysis			Multivariate analysis		
Variables		HR	95% CI	p-value	HR	95% CI	p-value
Age		1.025	0.958-1.097	0.469			
Sex		1.577	0.607-4.099	0.350			
Partial response on preoperative CT		5.331	0.213-133.434	0.308			
Adjacent vein resection		1.226	0.347-4.325	0.752			
Cell differentiation	WD,MD/PD,UD	59.675	5.955-597.789	0.001	86.399	5.806-1285.760	0.001
T-stage (AJCC 8th)	1,2/3,4	6.007	1.292-27.939	0.022	6.047	0.910-40.206	0.063
N-stage (AJCC 8th)	N0 (ref)	1		0.724			
	N1	1.623	0.433-6.089	0.473			
	N2	1.643	0.315-8.562	0.556			
Tumor regression grade	0,1/2,3	1.500	0.181-12.391	0.707			
Lymphovascular invasion		1.606	0.480-5.380	0.442			
Perineural invasion		1.612	0.191-13.583	0.661			
RDC		0.828	0.434-1.579	0.566			
R1 resection		3.861	1.171-12.728	0.026	3.776	0.990-14.403	0.052
Intraoperative transfusion		0.890	0.109-7.233	0.913			

HR, hazard ratio; CI, confidence interval; CT, computed tomography; WD, well differentiated; MD, moderately differentiated; PD, poorly differentiated; UD, undifferentiated; AJCC, American Joint Committee on Cancer; RDC, relative difference of CA19-9.

Table S6. Prognostic factors associated with recurrence free survival in patients with locally advanced pancreatic cancer and carbohydrate antigen 19-9 concentrations of 37–1000 U/ml before neoadjuvant chemotherapy (N = 60).

		Univariate analysis			Multivariate analysis		
**Variables**		**HR**	**95% CI**	**p-value**	**HR**	**95% CI**	**p-value**
Age		0.995	0.966-1.025	0.743			
Sex		0.990	0.612-1.601	0.966			
Partial response on preoperative CT		1.118	0.485-2.579	0.793			
Adjacent vein resection		1.605	0.803-3.207	0.180			
Cell differentiation	WD,MD/PD,UD	3.145	0.931-10.624	0.065			
Tumor regression grade	0,1/2,3	1.382	0.531-3.601	0.508			
Lymphovascular invasion		1.913	0.938-3.901	0.075			
Perineural invasion		1.417	0.582-3.451	0.442			
T-stage (AJCC 8th)	1,2/3,4	2.772	1.147-6.703	0.024	2.545	1.060-6.110	0.037
N-stage (AJCC 8th)	N0 (ref)			0.082			
	N1	1.958	0.894-4.289	0.093			
	N2	2.516	1.020-6.206	0.045			
RDC		0.633	0.460-0.872	0.005	0.634	0.451-0.889	0.008
R1 resection		1.695	0.780-3.685	0.183			
Intraoperative transfusion		0.793	0.185-3.392	0.754			

HR, hazard ratio; CI, confidence interval; CT, computed tomography; WD, well differentiated; MD, moderately differentiated; PD, poorly differentiated; UD, undifferentiated; AJCC, American Joint Committee on Cancer; RDC, relative difference of CA19-9.

Table S7. Prognostic performance of models that included decreases and normalization of carbohydrate antigen 19-9 concentration after neoadjuvant chemotherapy and surgery on overall survival and recurrence free survival for locally advanced pancreatic cancer.

Outcome		C-index	95% CI	p-value (1 vs. 2)	p-value (1 vs. 3)	AIC	95% CI	p-value (1 vs. 2)	p-value (1 vs. 3)
Overall survival	Model 1	0.919	0.812-1.000	0.972	0.616	49.475	14.014-73.311	0.944	0.892
	Model 2	0.907	0.764-0.989			49.327	15.632-73.872		
	Model 3	0.896	0.771-0.988			49.476	15.575-74.287		
Recurrence free survival	Model 1	0.646	0.553-0.767	0.932	0.908	193.838	134.477-241.639	0.992	0.992
	Model 2	0.606	0.521-0.717			199.314	140.454-246.144		
	Model 3	0.661	0.578-0.763			193.408	133.617-241.855		

CI, confidence interval; AIC, Akaike information criterion; Model 1, prognostic model including decreased carbohydrate antigen 19-9 concentration during neoadjuvant chemotherapy; Model 2, prognostic model including normalization of carbohydrate antigen 19-9 concentration after neoadjuvant chemotherapy; Model 3, prognostic model including normalization of carbohydrate antigen 19-9 concentration after surgery.
